# Least squares residual power series solutions for Kawahara and Rosenau-Hyman nonlinear wave interactions with applications in fluid dynamics

**DOI:** 10.1038/s41598-025-97639-3

**Published:** 2025-04-28

**Authors:** A. Hassan, A. A. M. Arafa, S. Z. Rida, M. A. Dagher, H. M. El Sherbiny

**Affiliations:** 1 Department of Science and Mathematical Engineering, Faculty of Petroleum and Mining Engineering, Suez University, Suez, Egypt; 2 Department of Mathematics, College of Science, Qassim University, Buraydah, Saudi Arabia; 3 Department of Mathematics, Faculty of Science, South Valley University, Qena, Egypt; 4 Faculty of Engineering, King Salman International University, El-Tor, Egypt; 5 Department of Mathematics and Computer Science, Faculty of Science, Suez University, Suez, Egypt

**Keywords:** Fractional derivatives, Least squares approximations, Residual power series method, Fractional Wronskian, Kawahara equation, Rosenau-Hyman equation, Numerical results, Mathematics and computing, Physics

## Abstract

The present study uses the least squares residual power series (LSRPS) method to obtain approximate solutions to the nonlinear fractional-order Kawahara and Rosenau- Hyman equations. This method combines the residual power series (RPS) technique and the least squares approach. The calculations are obtained using Caputo’s sense as a basis. To obtain approximations of solutions, the well-known RPS method is first used. The functions are then proven to be linearly independent by checking the Wronskian determinant at fractional order. Next, a system of linear equations is generated and processed using the least squares approach. Using the least squares method, which uses fewer expansion terms than the classical RPS method, approximate solutions are determined. The problems presented below demonstrate how much faster the proposed method converges compared to the RPS method. Numerical results are presented to demonstrate the efficiency, accuracy, and rapid convergence of the method.

## Introduction

Fractional calculus has grown in popularity and application in many fields of science and engineering over the last few decades, including fluid mechanics, diffusive transport, electrical networks, electromagnetic theory, and various branches of physics, biological sciences, and other applications^1–5^. When it comes to modeling physical events, using fractional derivatives instead of integer derivatives provides greater benefits. The fractional derivatives improve the model by incorporating memory effects and nonlocal interactions, making it more applicable to complex physical systems. Caputo’s derivative is among the most often used fractional derivatives because it allows initial conditions to be included in the same way as classical differential equations, which is crucial in physics and engineering^1^. The definition is also more important than other definitions because it has a better memory effect than other definitions. Caputo derivative has been widely studied and applied in engineering, physics, and control systems. The ABC derivative is still relatively new, which means that there are few validated numerical methods, tools, and practical applications. Most nonlinear differential equations are often produced by modeling various physical systems mathematically. Finding analytical solutions to these problems is typically exceedingly challenging. The Roseau-Hyman equation was discovered as a simplified model for studying the role of nonlinear dispersion in pattern creation in liquid droplets, and it has found several uses in the modeling of various issues in physics and engineering^6^. The fractional Kawahara equation is an extension of the classical Kawahara equation, a higher-order, nonlinear partial differential equation used to describe wave propagation in dispersive media. This equation also simulates the propagation of long waves in shallow water, plasma waves, and fluid dynamics in systems. In the past, this problem has been extensively studied using a variety of methods, see^7–10^. The Kawahara equation is crucial for explaining the kinematics of plasma waves, capillary-gravity water waves, surface tension water waves, shallow water waves, and other wave types^11–14^. The fractional Rosenau-Heymann (RH) equation is a generalization of the classical Rosenau-Heymann equation, which models nonlinear wave phenomena, such as localized waves that do not dissipate over time^15–18^. It also has physical applications in Shallow water waves, Plasma and optical pulse propagation. For various fractional differential equations, several approaches have been investigated. These include the Double (G’/G, 1/G)-expansion technique^19,20^, the Lie group analysis and Lie’s invariant analysis methods^21,22^, the Laguerre wavelet collocation method^23^, the fractional reduced differential transform method^24^, the Hirota bilinear method, the variational iteration method, Kudryashov method^25–29^, Adomian decomposition method^30^, Homotopy analysis method^31^, RPS method^32–34^ and other efficient techniques^35–44^ which are powerful tools to find the solutions of the nonlinear partial differential equations (NPDEs). The main objective of this study is to develop and validate semi-analytical solutions to the time-fractional Kawahara and Rosenau- Hyman equations arising in fluid dynamics using the LSRPS method with the Caputo definition of time-fractional derivatives. The study aims to demonstrate the advantages of the LSRPS method in dealing with nonlinear terms without assumptions and to demonstrate the effects of enhanced memory. The LSRPS method is computationally efficient and robust for solving both linear and nonlinear equations. By utilizing least squares minimization and iterative projection, this method offers a powerful alternative to traditional direct and iterative methods, especially in cases involving unconditional or large-scale problems.

The following is the paper’s structure: The definitions of Caputo and Wronskian’s fractional are introduced in Sect. 2. Section 3 suggests the LSRPS approach. Section 4 Displays some applications of LSRPS Method. There is a conclusion in Sect. 5.

## Preliminaries

Some basic concepts and properties of fractional calculus theory relevant to this section will be introduced. The Wronskian fractional equation is also described in this section.

### Riemann-Liouville fractional integral

The Riemann-Liouville fractional integral of order$\:\:\alpha\:>0$ of a function$\:\:f:\:{R}^{+}\to\:R$ is defined as:

1 $$J^{\alpha } f\left( x \right) = \frac{1}{{\Gamma \left( \alpha \right)}}\int\limits_{0}^{x} {\left( {x - t} \right)^{{\alpha \: - 1}} f\left( t \right)\;dt} ,\quad \alpha > 0,\:x > 0,$$

where

2 $$\:{J}^{0}f\left(x\right)=f\left(x\right).$$

Hence, we have:

3 $$J^{\alpha } t^{\gamma } = \frac{{\Gamma \left( {\gamma + 1} \right)}}{{\Gamma \left( {\alpha + \gamma + 1} \right)}}t^{{\alpha + \gamma }} .\quad \alpha > 0,\:\gamma > - 1,\:t > 0.$$

### Riemann-Liouville fractional derivative

Riemann-Liouville fractional derivatives of order$\:\:\alpha\:$ of a continuous function $\:f:\:{R}^{+}\to\:R\:$is obtained consecutively by:

4 $$\begin{gathered} D^{\alpha } f\left( x \right) = D^{m} \left( {J^{{m - \alpha }} f\left( x \right)} \right), \hfill \\ D_{{\text{*}}}^{\alpha } f\left( x \right) = J^{{m - \alpha }} \left( {D^{m} f\left( x \right)} \right), \hfill \\ \end{gathered}$$

where$$m - 1 < \alpha \le m,\:m \in N.$$

### The Caputo fractional derivative

The Caputo fractional derivative of a continuous function $\:f:\:{R}^{+}\to\:R$ may be represented as follows:

5 $$\:{\:D}^{\alpha\:}f\left(x\right)={J}^{m-\alpha\:}\left({D}^{m}f\left(x\right)\right)=\frac{1}{{\Gamma\:}(m+\alpha\:)}{\int\:}_{0}^{x}{\left(x-t\right)}^{m-\alpha\:-1}{f}^{m}\left(t\right)dt,$$

where $$m - 1 < \alpha \le m,\:m \in N,\:x > 0.$$

Some of the fundamental fractional derivatives and integrals for$\:\alpha ,\:\beta \in R^{ + }$ are as follows:

6 $$\begin{gathered} \:J^{{\alpha \:}} J^{{\beta \:}} \:f\left( x \right) = \:J^{{\alpha \: + \beta \:}} \:f\left( x \right), \hfill \\ \:J^{{\alpha \:}} J^{{\beta \:}} \:f\left( x \right) = \:J^{{\beta \:}} J^{{\alpha \:}} \:f\left( x \right) \hfill \\ \:J^{{\alpha \:}} t^{{\gamma \:}} \: = \:\frac{{\Gamma \:(\gamma \: + 1)}}{{\Gamma \:(\alpha \: + \gamma \: + 1)}}t^{{\alpha \: + \gamma \:}} ,\:\:\:\:\:\:\alpha > 0,\:\gamma > - 1,\:t > 0. \hfill \\ \end{gathered}$$

### Caputo fractional derivative characteristics

If $\: - 1 < \alpha \le m,\:m \in N,\:\mu \ge - 1\:$it holds:

7 $$\begin{gathered} 1.\quad D^{{\alpha \:}} J^{{\alpha \:}} f\left( x \right) = f\left( x \right), \hfill \\ 2.\quad J^{{\alpha \:}} \:D^{{\alpha \:}} f\left( x \right) = f\left( x \right) + \sum\limits_{{k = 0}}^{{m - 1}} {f^{k} \left( {0^{ + } } \right)\frac{{x^{k} }}{{k!}},} \:\:x > 0, \hfill \\ 3.\quad D^{{\alpha \:}} \left( C \right) = 0,\:C\:{\text{is}}\:{\text{constant}}. \hfill \\ 4.\quad D^{{\alpha \:}} \left( {\xi \:\:f\left( t \right) + \theta \:g\left( t \right)} \right) = \:\xi \:\:D^{{\alpha \:}} \left( {f\left( t \right)} \right) + \theta \:\:D^{{\alpha \:}} \left( {g\left( t \right)} \right). \hfill \\ \end{gathered}$$

where$\:\:\xi\:$,$\:\theta\:$, and C are real constants.

### **The fractional partial Wronskian** (see^45^)

Let $\:{\varphi\:}_{1},{\varphi\:}_{2},\dots\:,{\varphi\:}_{n}\:$are the number of functions of the variables x and t that are specified on the domain$\:\:{\Omega\:}$. Then, the fractional partial Wronskian of$\:{\:\varphi\:}_{1},{\varphi\:}_{2},\dots\:,{\varphi\:}_{n}$ take the form:

8 $$\begin{aligned} \: & \omega ^{\alpha } \left[ {\varphi _{1} ,\varphi _{2} , \ldots ,\varphi _{n} } \right] \\ & \quad = \left| {\begin{array}{*{20}l} {\varphi _{0} } \hfill & {\varphi _{1} } \hfill & {\varphi _{2} } \hfill & \cdots \hfill & {\varphi _{n} } \hfill \\ {D^{\alpha } \left( {\varphi _{0} } \right)} \hfill & {D^{\alpha } \left( {\varphi _{1} } \right)} \hfill & {D^{\alpha } \left( {\varphi _{2} } \right)} \hfill & \cdots \hfill & {D^{\alpha } \left( {\varphi _{n} } \right)} \hfill \\ {D^{{2\alpha }} \left( {\varphi _{0} } \right)} \hfill & {D^{{2\alpha }} \left( {\varphi _{1} } \right)} \hfill & {D^{{2\alpha }} \left( {\varphi _{2} } \right)} \hfill & \cdots \hfill & {D^{{2\alpha }} \left( {\varphi _{n} } \right)} \hfill \\ \vdots \hfill & \vdots \hfill & \vdots \hfill & \vdots \hfill & \vdots \hfill \\ {D^{{\left( {n - 1} \right)\alpha }} \left( {\varphi _{0} } \right)} \hfill & {D^{{\left( {n - 1} \right)\alpha }} \left( {\varphi _{1} } \right)} \hfill & {D^{{\left( {n - 1} \right)\alpha }} \left( {\varphi _{2} } \right)} \hfill & \cdots \hfill & {D^{{\left( {n - 1} \right)\alpha }} \left( {\varphi _{n} } \right)} \hfill \\ \end{array} } \right| \ne 0, \\ \end{aligned}$$

where $$\:0<\alpha\:\le\:1,{\:D}^{\alpha\:}\left({\varphi\:}_{i}\right)=\left(\frac{\partial\:}{\partial\:x}+\frac{{\partial\:}^{\alpha\:}}{{\partial\:t}^{\alpha\:}}\right)\left({\varphi\:}_{i}\right),\:\text{w}\text{h}\text{e}\text{r}\text{e}\:i=1,\:2,\:3,\dots\:,n.$$

$\:{\:D}^{n\alpha\:}={\:D}^{\alpha\:}{\:D}^{\alpha\:}\dots\:{\:D}^{\alpha\:}\left(\:n-times\right)$and $\:{\varphi\:}_{1}\left(x.t\right),{\varphi\:}_{2}\left(x.t\right),\dots\:,{\varphi\:}_{n}(x.t)\:$ are considered to be linearly independent if and only if the fractional partial Wronskian of all n functions$\:{\:\varphi\:}_{1}\left(x.t\right),{\varphi\:}_{2}\left(x.t\right),\dots\:,{\varphi\:}_{n}(x.t)\:$is nonzero at least once in the domain$\:\:{\Omega\:}\:$=$\:\left[a.b\right]\times\:[a.b]$.

### Theorem (Convergence theorem) (see^46–48^)

Suppose that $$\:{\text{u}}\left( {{\text{x,t}}} \right) \in {\text{C}}\left( {\left[ {{\text{r,t}}_{0} } \right] \times \:\left[ {{\text{r,t}}_{0} + {\text{r}}} \right]} \right),\:{\text{D}}_{{\text{t}}}^{{{\text{i}}\upalpha }} {\text{u}}\left( {{\text{x,t}}} \right) \in {\text{C}}\left( {\left[ {{\text{r,t}}_{0} } \right] \times \:\left[ {{\text{r,t}}_{0} + {\text{r}}} \right]} \right)$$

$$\:0 \le \:{\text{m}} - 1 \le \:\alpha \: \le \:{\text{m}}\:{\text{and}}\:{\text{D}}_{{\text{t}}}^{{{\text{i}}\upalpha }} {\text{u}}\left( {{\text{x,t}}} \right)\:{\text{can}}\:{\text{be}}\:{\text{for}}\:{\text{i}} = 0,1,2, \ldots {\text{,N}} + 1\:{\text{where}}$$

with respect to t on ($\:\:{\text{t}}_{0} {\text{,t}}_{0} + {\text{r}})$ then $\:\text{m}-1$ differentiate

$$\:{\text{u}}\left( {{\text{x,t}}} \right) \cong \:\sum {_{{{\text{j}} = 0}}^{{{\text{m}} - 1}} } \sum {_{{{\text{i}} = 0}}^{{\text{N}}} } {\text{U}}_{{{\text{j}} + {\text{i}}\upalpha \:}} \left( {\text{x}} \right)\left( {{\text{t}} - {\text{t}}_{0} } \right)^{{{\text{j}} + {\text{i}}\upalpha \:}} ,$$

where

$$\:{\text{U}}_{{{\text{j}} + {\text{i}}\upalpha }} \left( {\text{x}} \right) = \frac{{{\text{D}}_{{\text{t}}}^{{{\text{i}}\upalpha \: + {\text{J}}}} }}{{\Gamma \:({\text{i}}\upalpha + {\text{j}} + 1)}}{\text{u}}\left( {{\text{x,t}}_{0} } \right),$$

and $\:\text{r}$ is the radius of convergence. Moreover, the error term $\:{\text{r}}_{{\text{N}}} \left( {{\text{x,t}}} \right)\:{\text{has}}\:{\text{the}}\:{\text{form}}$

$$\:\left\| {{\text{r}}_{{\text{N}}} \left( {{\text{x,t}}} \right)} \right\| = {\text{sup}}_{{{\text{t}} \in \left[ {0{\text{,T}}} \right]}} \left| {\sum\limits_{{{\text{j}} = 0}}^{{{\text{m}} - 1}} {\left( {\frac{{{\text{D}}^{{({\text{N}} + 1)\alpha + {\text{j}}}} {\text{u}}\left( {{\text{x}},\upxi } \right){\text{t}}^{{({\text{N}} + 1)\alpha + {\text{j}}}} }}{{\Gamma (({\text{N}} + 1)\upalpha + {\text{i}} + 1)}}} \right)} } \right|,\:{\text{where}}\:0 \le \upxi \le {\text{t}}.$$

Proof (see^48^).

## Methodology

In this section, we will outline the general procedure of the LSRPS technique, as described in^46,47^ for solving time-fractional differential equations. This technique combines the classical (RPS) method with the least-squares method.

### Residual power series method (RPSM)

Considering the following time-fractional differential equation:

9 $$\:{l}^{\alpha\:}\left(u\left(x,t\right)\right)+N\left(u\left(x,t\right)\right)=0,\:\:t>0,\:\:0<\alpha\:\le\:1,$$

where $\:{l}^{\alpha\:}$ is a linear fractional operator,$\:\:N$ is a nonlinear operator, *u* (*x*,* t*) is an unknown function, and *I* is an initial condition. Based on the classical (RPS) technique as described in references^32–34^, an algorithm can be proposed as follows:

10 $$\:u\left( {x,t} \right) = \sum\limits_{{n = 0}}^{\infty } {f_{n} \left( {\text{x}} \right)\frac{{t^{{n\upalpha }} }}{{\Gamma (1 + {\text{n}}\alpha )}}} ,\:0 < \alpha \le 1,\:x \in I,\:0 \le t < R.$$

To obtain a reasonable approximation for Eq. (9), the K^th^ series of$\:\:u\left(x.t\right)$ is introduced. Thus, the truncated series$\:\:{\:u}_{k}(x.t)$ is defined as follows

11 $$\:u_{k} \left( {x,t} \right) = \sum\limits_{{n = 0}}^{\infty } {} f_{n} \left( {\text{x}} \right)\frac{{t^{{n\upalpha }} }}{{\Gamma (1 + {\text{n}}\alpha )}},\:0 < \alpha \le 1,\:x \in I,\:0 \le t < R.$$

The 0-th RPS approximate solution of $\:\:u\left(x.t\right)\:$is:

12 $$\:{u}_{0}\left(x,t\right)=\:u\left(x,0\right)=\:f\left(x\right).$$

Equation (9) can be written by:

13 $$\:u_{k} \left( {x,t} \right) = f\left( x \right) + \sum\limits_{{n = 1}}^{\infty } {f_{n} \left( {\text{x}} \right)\frac{{t^{{n\upalpha \:}} }}{{\Gamma (1 + {\text{n}}\alpha )}}} ,\:0 < \alpha \le 1,\:x \in I,\:0 \le t < R.$$

Let’s define the residual function for Eq. (9) as follows:

14 $$\:{{Res}_{u}\left(x,t\right)=l}^{\alpha\:}\left({u}_{k}\left(x,t\right)\right)+N\left({u}_{k}\left(x,t\right)\right),\:\:t>0,\:\:0<\alpha\:\le\:1.$$

Considering the initial condition$\:\:I\left(u\right)=0$, let’s define the K^th^ residual function $\:{Res}_{u.k}$ as follows:

15 $$\:{{Res}_{u.k}\left(x,t\right)=l}^{\alpha\:}\left({u}_{k}\left(x,t\right)\right)+N\left({u}_{k}\left(x,t\right)\right),\:\:t>0,\:\:0<\alpha\:\le\:1.$$

To obtain $\:f_{n} \left( {\text{x}} \right),\:n \in N^{*} ,$ we seek the solution of the following Eq. 

16 $$\:D_{t}^{{(n - 1)\alpha \:}} Res_{{u.k}} \left( {x,0} \right) = 0,\:\:k \in N^{{\text{*}}} ,$$

where $$\:{N}^{*}=\left\{1,\:2,\:3,.\dots\:,n\right\}.$$

To determine$\:{f}_{1}\left(\text{x}\right)$,$\:\:{f}_{2}\left(\text{x}\right),{f}_{3}\left(\text{x}\right)$, …, we consider $\:(k=1,\:2,\:3.\dots\:.)$ in Eq. (10) and substitute this series expansion into Eq. (9) to obtain an approximate solution for Eq. (1). The standard residual power series approach can be employed to obtain K^th^ order approximation solutions as:

17 $$\:{u}_{k}={\varphi\:}_{0}+{\varphi\:}_{1}+{\varphi\:}_{2\:}+\dots\:{\varphi\:}_{k},$$

where,

18 $$\begin{array}{*{20}l} {\varphi _{0} = f_{0} \left( {\text{x}} \right),} \hfill \\ {\varphi _{1} = f_{1} \left( {\text{x}} \right)\frac{{t^{{\alpha \:}} }}{{\Gamma \left( {1 + \upalpha } \right)}},} \hfill \\ {\varphi _{2} = f_{2} \left( {\text{x}} \right)\frac{{t^{{2\alpha }} }}{{\Gamma \left( {1 + 2\upalpha } \right)}},} \hfill \\ \vdots \hfill \\ {\varphi _{k} = f_{k} \left( {\text{x}} \right)\frac{{t^{{k\alpha }} }}{{\Gamma (1 + {\text{k}}\upalpha )}}.} \hfill \\ \end{array}$$

### Least-Squares residual power series technique (LSRPS)

This section presents the methodology for the LSRPS technique and introduces key definitions necessary for its implementation.

Let the remainder $\:\widetilde{Res}$ for Eq. (1) be:

19 $$\:\widetilde{{Res}}\left( {x,t,\widetilde{u}} \right) = \:l^{{\alpha \:}} ({\text{u}}\:~\left( {x,t} \right)) + N\left( {\widetilde{u}\left( {x,t} \right)} \right),\:\:t > 0,\:\:0 < \alpha \le 1,$$

with$\:\:I\left(\widetilde{u}\right)=0$, and $\:\widetilde{u}$ is the approximate solution of Eq. (2).

Remark that if

20 $$\:\underset{i\to\:{\infty\:}}{\text{lim}}\widetilde{Res}\left(x,t,{s}^{i\alpha\:}(x,t)\right)=0,$$

where $\:{{\{s}^{i\alpha\:}(x,t)\}\:\:}_{i\in\:{N}^{*}}\:\:$is converge to the solution of Eq. (1).

The $\:\widetilde{u}$ is the $\:\epsilon\:-$approximate RPS method solution of Eq. (1) on domain $\:{\Omega\:}$ if:

21 $$\:\left|\widetilde{Res}\left(x,t,\widetilde{u}\right)\right|<\:\epsilon\:,$$

and $$\:\:I\left(u\right)=0\:\text{i}\text{s}\:\text{a}\text{l}\text{s}\text{o}\:\text{s}\text{a}\text{t}\text{i}\text{s}\text{f}\text{i}\text{e}\text{d}\:\text{b}\text{y}\:\widetilde{u}$$.

If $\:\widetilde{u}$ is the weak -approximate (RPS) method solution of Eq. (1) on domain$\:\:{\Omega\:}$, we call it that:

22 $$\:\iint\:{\widetilde{Res}\left(x,t,\widetilde{u}\right)}^{2}\:dx\:dt\le\:\epsilon\:,$$

where$$\:I\left(u\right)=0\:\text{i}\text{s}\:\text{a}\text{l}\text{s}\text{o}\:\text{s}\text{a}\text{t}\text{i}\text{s}\text{f}\text{i}\text{e}\text{d}\:\text{b}\text{y}\:\widetilde{u}$$.

To implement the least-squares Residual Power Series approach, we propose the following procedures:

### 1st step

We adopt the classical residual power series approach to approximate the solution. The expression for $\:{u}_{k}\left(x.t\right)$ can be represented as follows:

23 $$\:u_{k} \left( {x,t} \right) = \sum \: _{{n = 0}}^{{\infty \:}} f_{n} \left( {\text{x}} \right)\frac{{t^{{n\upalpha \:}} }}{{\Gamma (1 + {\text{n}}\alpha )}},\:\:0 < \alpha \le 1,\:x \in I,\:0 \le t < R,$$

and the k^th^ residual function $\:{Res}_{u.k}$ take the form:

24 $$\:{{Res}_{u.k}\left(x,t\right)=l}^{\alpha\:}\left({u}_{k}\left(x,t\right)\right)+N\left({u}_{k}\left(x,t\right)\right),\:\:t>0,\:\:0<\alpha\:\le\:1.$$

Subsequently, we seek solutions for $\:{f}_{n}\left(\text{x}\right)$ by exploring the following procedure:

25 $$\:{D}_{t}^{(n-1)\alpha\:}{Res}_{u.k}\left(x,0\right)=0.\:k\in{N}^{\text{*}},$$

where $$\:{N}^{*}=\{1,\:2,\:3,.,n\}.$$

In this case, the implementation of the RPS technique provides kth-order approximation solutions characterized by the following:

26 $$\:{u}_{k}={\varphi\:}_{0}+{\varphi\:}_{1}+{\varphi\:}_{2\:}+\dots\:{\varphi\:}_{k\:},$$

where $\:{\varphi\:}_{0},{\varphi\:}_{1},{\varphi\:}_{2\:}$can be computed by Eq. (4)

### 2nd step

The linearly independent functions can be verified or validated using the following procedure:

27 $$\begin{aligned} & \:\omega \:^{{\alpha \:}} \left[ {\varphi \:_{1} .\varphi \:_{2} . \ldots \:.\varphi \:_{n} } \right] \\ & \quad = \left| {\begin{array}{*{20}l} {\varphi _{0} } \hfill & {\varphi _{1} } \hfill & {\varphi _{2} } \hfill & \ldots \hfill & {\varphi _{n} } \hfill \\ {D^{\alpha } \left( {\varphi _{0} } \right)} \hfill & {D^{{\alpha \:}} \left( {\varphi \:_{1} } \right)} \hfill & {D^{{\alpha \:}} \left( {\varphi \:_{2} } \right)} \hfill & \ldots \hfill & {D^{{\alpha \:}} \left( {\varphi \:_{n} } \right)} \hfill \\ {D^{{2\alpha }} \left( {\varphi _{0} } \right)} \hfill & {D^{{2\alpha \:}} \left( {\varphi \:_{1} } \right)} \hfill & {D^{{2\alpha \:}} \left( {\varphi \:_{2} } \right)} \hfill & \cdots \hfill & {D^{{2\alpha \:}} \left( {\varphi \:_{n} } \right)} \hfill \\ \vdots \hfill & \vdots \hfill & \vdots \hfill & {} \hfill & \vdots \hfill \\ {D^{{\left( {n - 1} \right)\alpha \:}} \left( {\varphi \:_{0} } \right)} \hfill & {D^{{\left( {n - 1} \right)\alpha \:}} \left( {\varphi \:_{1} } \right)} \hfill & {D^{{\left( {n - 1} \right)\alpha \:}} \left( {\varphi \:_{2} } \right)} \hfill & \cdots \hfill & {D^{{\left( {n - 1} \right)\alpha \:}} \left( {\varphi \:_{n} } \right)} \hfill \\ \end{array} } \right| \ne \:0, \\ \end{aligned}$$

where $\:{\:D}^{\alpha\:}\left({\varphi\:}_{i}\right)=\left(\frac{\partial\:}{\partial\:x}+\frac{{\partial\:}^{\alpha\:}}{{\partial\:t}^{\alpha\:}}\right)\left({\varphi\:}_{i}\right),\:$and $\:{s}_{k}=\{{\varphi\:}_{0},{\varphi\:}_{1},\dots\:,{\varphi\:}_{n}\}$ be a set of linearly independent elements in the vector space of continuous functions defined on R.

If it is not possible to identify any point where $\:{\omega\:}^{\alpha\:}\left[{\varphi\:}_{1},{\varphi\:}_{2},\dots\:,{\varphi\:}_{n}\right]$ is not equal to 0, then it implies that the set of functions$\:\:{s}_{k}$ is linearly dependent.

### 3rd step

We assume that:

28 $$\:{\widetilde{u}}_{k}=\sum\:_{n=0}^{k}{c}_{k}^{n}\:{\varphi\:}_{r}.$$

By considering the approximated solution$\:\:{\widetilde{\text{u}}}_{\text{k}}$ for Eq. (1), we can substitute it into Eq. (5) to obtain:

29 $$\:\widetilde{Res}\left(x,t,{c}_{k}^{n}\right)=\widetilde{Res}\left(x,t,{\widetilde{u}}_{k}\right).$$

### 4th step

We relate the following functional to:

30 $$\:\int \: \int \: _{{\Omega \:}} \:(\widetilde{{Res}}\left( {x,t,\widetilde{u}} \right))^{2} \:dx\:dt\: = min\:J,$$

and obtain some constants of$\:\:{c}_{n}$ by solving the algebraic systems $\:\frac{{\partial \:J}}{{\partial \:c_{n} }} = 0,\:n = 1{\text{,}}2{\text{,}} \ldots ,k$.

### Application of the least-squares residual power series method

this section focuses on the application of the LSRPS method to address various problems. During the initial iterations of this new strategy, we often utilize the fractional RPS technique. The unidentified coefficients are subsequently determined using the least-squares method. To assess the accuracy of the approximation solutions, we employ graphs and tables, providing a visual and numerical analysis.

#### Problem 1

Considering the time-fractional Rosenau-Hyman equation:

31 $$\:{\text{D}}_{\text{t}}^{{\upalpha\:}}u-3{u}_{xx}{u}_{x}-u{u}_{x}-u{u}_{xxx}=0,$$

where *t* >0,$$\:\:x\in\:R$$, 0 < *α* ≤ 1.

Subject to the initial condition:

32 $$\:u\left(x,0\right)=-\frac{8\:\text{c}}{3}{cos}^{2}\left(\frac{\text{x}}{4}\right)$$.

The exact solution at *α*=1 is

$$\:u\left(x,t\right)=-\frac{8\text{c}}{3}{cos}^{2}\left(\frac{\text{x}-\text{c}\text{t}}{4}\right)$$.

To introduce the solution of fractional Rosenau-Hyman equation, we can employ the well-known (RPS) method^34^ which offers a solution for the equation as:

33 $$\:u\left(x,t\right)=f\left(x\right)+{f}_{1}\left(\text{x}\right)\frac{{t}^{\alpha\:}}{ \Gamma \left(1+{\upalpha\:}\right)}+{f}_{2}\left(x\right)\frac{{t}^{2\alpha\:}}{ \Gamma \left(1+2{\upalpha\:}\right)}+{f}_{3}\left(x\right)\frac{{t}^{3\alpha\:}}{ \Gamma \left(1+3{\upalpha\:}\right)}+\dots\:$$

where

34 $$\:f\left(x\right)=-\frac{8\text{c}}{3}{cos}^{2}\left(\frac{\text{x}}{4}\right),$$

35 $$\:{f}_{1}\left(x\right)=\frac{-2{c}^{2}}{3}\text{sin}\left(\frac{\text{x}}{2}\right),$$

36 $$\:{f}_{2}\left(x\right)=\frac{{c}^{3}}{3}\text{c}\text{o}\text{s}\left(\frac{\text{x}}{2}\right).$$

The linearly independent functions could be validated by using:

37 $$\begin{aligned} & \:\omega \:^{{\alpha \:}} \left[ {\varphi \:_{0} ,\varphi \:_{1} , \ldots \:,\varphi \:_{n} } \right] \\ & \quad = \left| {\begin{array}{*{20}c} { - \frac{{8{\text{c}}}}{3}cos^{2} \left( {\frac{{\text{x}}}{4}} \right).} \\ {\:\:D^{{\alpha \:}} \left( { - \frac{{8{\text{c}}}}{3}cos^{2} \left( {\frac{{\text{x}}}{4}} \right).} \right)} \\ {\begin{array}{*{20}c} {\:D^{{2\alpha \:}} \left( { - \frac{{8{\text{c}}}}{3}cos^{2} \left( {\frac{{\text{x}}}{4}} \right).} \right)} \\ \vdots \\ {\:\:D^{{\left( {n - 1} \right)\alpha \:}} \left( { - \frac{{8{\text{c}}}}{3}cos^{2} \left( {\frac{{\text{x}}}{4}} \right)} \right)} \\ \end{array} } \\ \end{array} \:\:\:\begin{array}{*{20}c} {\:\left( {\frac{{ - 2c^{2} }}{3}{\text{sin}}\left( {\frac{{\text{x}}}{2}} \right)} \right)} & {\left( {\frac{{c^{3} }}{3}{\text{cos}}\left( {\frac{{\text{x}}}{2}} \right)} \right)\:} & { \ldots \:} \\ {\:\:D^{{\alpha \:}} \left( {\frac{{ - 2c^{2} }}{3}{\text{sin}}\left( {\frac{{\text{x}}}{2}} \right))\frac{{t^{{\alpha \:}} }}{{\Gamma \left( {1 + \alpha \:} \right)}}} \right)} & {D^{{\alpha \:}} \left( {\left( {\frac{{c^{3} }}{3}{\text{cos}}\left( {\frac{{\text{x}}}{2}} \right)} \right)\frac{{t^{{2\alpha \:}} }}{{\Gamma \left( {1 + 2\alpha \:} \right)}}} \right)} & { \ldots \:} \\ {\:\begin{array}{*{20}c} {\:D^{{2\alpha \:}} \left( {\frac{{ - 2c^{2} }}{3}{\text{sin}}\left( {\frac{{\text{x}}}{2}} \right))\frac{{t^{{\alpha \:}} }}{{\Gamma \left( {1 + \alpha \:} \right)}}} \right)} \\ \vdots \\ {\:\:D^{{\left( {n - 1} \right)\alpha \:}} \:\left( {\frac{{ - 2c^{2} }}{3}{\text{sin}}\left( {\frac{{\text{x}}}{2}} \right)} \right)} \\ \end{array} } & {\begin{array}{*{20}c} {\:D^{{2\alpha \:}} \left( {\left( {\frac{{c^{3} }}{3}{\text{cos}}\left( {\frac{{\text{x}}}{2}} \right)} \right)\frac{{t^{{2\alpha \:}} }}{{\Gamma \left( {1 + 2\alpha \:} \right)}}} \right)} \\ \vdots \\ {\:\:D^{{\left( {n - 1} \right)\alpha \:}} \:\left( {\frac{{c^{3} }}{3}{\text{cos}}\left( {\frac{{\text{x}}}{2}} \right)} \right)} \\ \end{array} } & { \ldots \:} \\ \end{array} } \right| \ne 0, \\ \end{aligned}$$

where $$\:\alpha\:=1,\:t=0.5,\:x=0,c=1\:\text{a}\text{n}\text{d}\:{\omega\:}^{1}\left[{\varphi\:}_{0},{\varphi\:}_{1},{\varphi\:}_{2}\right]\ne\:0.\:\:$$

Hence, the functions $\:{\varphi\:}_{0},{\varphi\:}_{1},{\varphi\:}_{2}$ are linearly independent define as:

$$\:{\varphi\:}_{0}=f\left(x\right),$$

$$\:{\varphi\:}_{1}={f}_{1}\left(x\right)\frac{{t}^{\alpha\:}}{ \Gamma \left(1+\alpha\:\right)},$$

$$\:{\varphi\:}_{2}={f}_{2}\left(x\right)\frac{{t}^{2\alpha\:}}{ \Gamma \left(1+2\alpha\:\right)}.$$

Consequently, we can obtain an approximation that can be formulated as follows:

38 $$\:\widetilde{u}={\text{c}}_{0}\left(-\frac{8}{3}{cos}^{2}\left(\frac{\text{x}}{4}\right)\right)+{\text{c}}_{1}\left(\left(\frac{-2}{3}\text{sin}\left(\frac{\text{x}}{2}\right)\right)\right)\frac{{\text{t}}^{{\upalpha\:}}}{ \Gamma \left(1+{\upalpha\:}\right)}+{\text{c}}_{2}\left(\left(\frac{1}{3}\text{cos}\left(\frac{\text{x}}{2}\right)\right)\right)\frac{{\text{t}}^{2{\upalpha\:}}}{ \Gamma \left(1+2{\upalpha\:}\right)}.$$

The residual function can be obtained by:

39 $$\:\widetilde{Res}\left(x,t,\widetilde{u}\right)={\text{D}}_{\text{t}}^{{\upalpha\:}}\widetilde{u}-\widetilde{u}{\widetilde{u}}_{x}-3\left({\widetilde{u}}_{x}\right){\widetilde{u}}_{xx}-\widetilde{u}{\widetilde{u}}_{xxx}.$$

With the initial condition:

40 $$\:{\widetilde{u}}_{0}=-\:{c}_{0}\left(\frac{8}{3}{cos}^{2}\left(\frac{\text{x}}{4}\right)\right).$$

By using $\:{\widetilde{u}}_{0}$ put $\:{c}_{0}=1,\:\:\widetilde{u}\:$can be written as:

41 $$\:\widetilde{u}=\:(\frac{8}{3}{cos}^{2}\left(\frac{\text{x}}{4}\right)+{\text{c}}_{1}\left((\frac{-2}{3}\text{s}\text{i}\text{n}(\frac{\text{x}}{2})\right)\frac{{\text{t}}^{{\upalpha\:}}}{ \Gamma \left(1+{\upalpha\:}\right)}+{\text{c}}_{2}\left(\left(\frac{1}{3}\text{c}\text{o}\text{s}\right(\frac{\text{x}}{2}\left)\right)\right)\frac{{\text{t}}^{2{\upalpha\:}}}{ \Gamma \left(1+2{\upalpha\:}\right)}.$$

By substituting $\:\widetilde{u}$ into $\:\widetilde{Res}\left(x,t,\widetilde{u}\right)$, we can obtain$\:\:\widetilde{Res.}$ As a result, the functional J can be expressed as:

42 $$\:{\iint\:}_{{\Omega\:}}^{.}{\widetilde{Res}\left(x,t,\widetilde{u}\right)}^{2}\:dx\:dt=\:J\left({c}_{1},{c}_{2}\right).$$

We have two algebraic equations:

43 $$\:\frac{\partial\:J}{\partial\:{c}_{1}}=0,\:\frac{\partial\:J}{\partial\:{c}_{2}}=0.$$

And following that, we calculate the unknown coefficients of Eq. (43) when$\:\:\alpha\:=1$ as:

44 $$\:{c}_{1}=0.997977578799891,{c}_{2}=1.013022733315692.$$

The absolute error between the exact and approximated solutions using the proposed technique can be illustrated using the following formula:

45 $$\:Error=\left|{\widetilde{u}}_{i}(x,t)-u(x,t)\right|.$$

#### Problem 2

We will now examine the fractional Kawahara Eq. 

46 $$\:{\text{D}}_{\text{t}}^{{\upalpha\:}}u+\left(u\right){u}_{x}+{u}_{xxx}-{u}_{xxxxx}=0,$$

where *t* >0, $$\:x\in\:R$$, 0 < *α* ≤ 1.

Subject to the initial condition:

47 $$\:u\left(x,0\right)=f\left(x\right)=\frac{105}{169}\:{sech}^{4}\left(\frac{x}{2\:\sqrt{13}}\right).$$

The exact solution when$\:\:{\upalpha\:}=1$ is:

48 $$\:u\left(x,t\right)=\frac{105}{169}\:{{sech}}^{4}\left[\frac{1}{2\:\sqrt{13}}(x-\frac{36\:t}{169})\right].$$

By employing a similar approach as the classical residual power series method demonstrated in problem (1), we can derive the following Eq. 

49 $$\:{\text{f}}_{1}\left(\text{x}\right)=\:\frac{7560\:\:{\text{s}\text{e}\text{c}\text{h}}^{4}\left(\frac{\text{x}}{2\sqrt{13}\:}\right)\text{tanh}\left(\frac{\text{x}}{2\sqrt{13}\:}\right)}{28561\:\sqrt{13}},$$

50 $$\:{f}_{2}\left(x\right)=\:\:\frac{136080\:\:{sech}^{6}\left(\frac{x}{2\sqrt{13}\:}\right)(-3+2\text{cosh}\left(\frac{x}{2\sqrt{13}\:}\right))}{62748517\:},$$

51 $$\:{f}_{3}\left(x\right)=\:\:\frac{4898880\:\:{sech}^{7}\left(\frac{x}{2\sqrt{13}\:}\right)(-13\text{\:sinh}(\frac{x}{2\sqrt{13}\:})+2\text{sinh}\left(\frac{x}{2\sqrt{13}\:}\right))}{10604499373\:\sqrt{13}\:},$$

52 $$\:{f}_{4}\left(x\right)=\:\:\frac{88179840\:\:{sech}^{8}\left(\frac{x}{2\sqrt{13}\:}\right)(52-49\text{\:cosh}(\frac{x}{2\sqrt{13}\:})+4\text{cosh}\left(\frac{2x}{\sqrt{13}\:}\right))}{23298085122481}.$$

The series representation of the solution is provided as follows:

53 $$\:u\left(x,t\right)=f\left(x\right)+{f}_{1}\left(\text{x}\right)\frac{{t}^{\alpha\:}}{ \Gamma \left(1+{\upalpha\:}\right)}+{f}_{2}\left(x\right)\frac{{t}^{2\alpha\:}}{ \Gamma \left(1+2{\upalpha\:}\right)}+{f}_{3}\left(x\right)\frac{{t}^{3\alpha\:}}{ \Gamma \left(1+3{\upalpha\:}\right)}+{f}_{4}\left(x\right)\frac{{t}^{4\alpha\:}}{ \Gamma \left(1+4{\upalpha\:}\right)}+ \cdots\:\:\:$$

To verify the linear independence of the functions, we can employ the following procedure:

54 $$\begin{aligned} & \:\omega \:^{{\alpha \:}} \left[ {\varphi _{0} ,\varphi \:_{1} , \ldots \:,\varphi \:_{n} } \right] \\ & \quad = \left| {\begin{array}{*{20}l} {\varphi _{0} } \hfill & {\varphi _{1} } \hfill & {\varphi _{2} } \hfill & {\varphi _{3} } \hfill & {\varphi _{4} } \hfill \\ {D^{\alpha } (\varphi _{0} )} \hfill & {D^{\alpha } (\varphi _{1} )} \hfill & {D^{\alpha } (\varphi _{2} )} \hfill & {D^{\alpha } (\varphi _{3} )} \hfill & {D^{\alpha } (\varphi _{4} )} \hfill \\ {D^{{2\alpha }} (\varphi _{0} )} \hfill & {D^{{2\alpha }} (\varphi _{1} )} \hfill & {D^{{2\alpha }} (\varphi _{2} )} \hfill & {D^{{2\alpha }} (\varphi _{3} )} \hfill & {D^{{2\alpha }} (\varphi _{4} )} \hfill \\ \vdots \hfill & \vdots \hfill & \vdots \hfill & \vdots \hfill & \vdots \hfill \\ {D^{{(n - 1)\alpha }} (\varphi _{0} )} \hfill & {D^{{(n - 1)\alpha }} (\varphi _{1} )} \hfill & {D^{{(n - 1)\alpha }} (\varphi _{2} )} \hfill & {D^{{(n - 1)\alpha }} (\varphi _{3} )} \hfill & {D^{{(n - 1)\alpha }} (\varphi _{4} )} \hfill \\ \end{array} } \right| \ne 0, \\ \end{aligned}$$

By evaluating the given parameters$\:\:\alpha\:=1,\:t=0.5\:\text{a}\text{n}\text{d}\:x=0$, we can determine that$\:\:\:{\omega\:}^{1}\left[{\varphi\:}_{0},{\varphi\:}_{1},{\varphi\:}_{2},{\varphi\:}_{3},{\varphi\:}_{4}\right]\ne\:0$. Consequently, it can be concluded that the functions $\:{\varphi\:}_{0}.{\varphi\:}_{1}.{\varphi\:}_{2}.{\varphi\:}_{3}.{\varphi\:}_{4}$ are linearly independent. These functions are defined as:

$$\:{\varphi\:}_{0}=f\left(x\right),$$

$$\:{\varphi\:}_{1}={f}_{1}\left(x\right)\frac{{t}^{\alpha\:}}{ \Gamma \left(1+\alpha\:\right)},$$

$$\:{\varphi\:}_{2}={f}_{2}\left(x\right)\frac{{t}^{2\alpha\:}}{ \Gamma \left(1+2\alpha\:\right)},$$

$$\:{\varphi\:}_{3}={f}_{3}\left(x\right)\frac{{t}^{3\alpha\:}}{ \Gamma \left(1+3\alpha\:\right)},$$

$$\:{\varphi\:}_{4}={f}_{4}\left(x\right)\frac{{t}^{4\alpha\:}}{ \Gamma \left(1+4\alpha\:\right)}.$$

Therefore, based on this observation, we can deduce an approximation that can be expressed as follows:

55 $$\begin{aligned} \widetilde{u} & = c_{0} \left( {\frac{{105}}{{169}}\:sech^{4} \left( {\frac{x}{{2\:\sqrt {13} }}} \right)} \right) + c_{1} \left( {\frac{{7560\:\:sech^{4} \left( {\frac{x}{{2\sqrt {13} \:}}} \right){\text{tanh}}\left( {\frac{x}{{2\sqrt {13} \:}}} \right)}}{{28561\:\sqrt {13} }}} \right)\frac{{t^{{\alpha \:}} }}{{\Gamma \left( {1 + \upalpha } \right)}} \\ & \quad + \:\:c_{2} \left( {\frac{{136080\:\:sech^{6} \left( {\frac{x}{{2\sqrt {13} \:}}} \right)\left( { - 3 + 2{\text{cosh}}\left( {\frac{x}{{2\sqrt {13} \:}}} \right)} \right)}}{{62748517\:}}} \right)\frac{{t^{{2\alpha \:}} }}{{\Gamma \left( {1 + 2\upalpha } \right)}} \\ & \quad + \:\:\:c_{3} \left( {\frac{{4898880\:\:sech^{7} \left( {\frac{x}{{2\sqrt {13} \:}}} \right)\left( { - 13\:{\text{sinh}}\left( {\frac{x}{{2\sqrt {13} \:}}} \right) + 2{\text{sinh}}\left( {\frac{x}{{2\sqrt {13} \:}}} \right)} \right)}}{{10604499373\:\sqrt {13} \:}}} \right)\frac{{t^{{3\alpha \:}} }}{{\Gamma \left( {1 + 3\upalpha } \right)}} \\ & \quad + c_{4} \left( {\frac{{88179840\:\:sech^{8} \left( {\frac{x}{{2\sqrt {13} \:}}} \right)\left( {52 - 49\:{\text{cosh}}\left( {\frac{x}{{2\sqrt {13} \:}}} \right) + 4{\text{cosh}}\left( {\frac{{2x}}{{\sqrt {13} \:}}} \right)} \right)}}{{23298085122481}}} \right)\frac{{t^{{4\alpha }} }}{{\Gamma \left( {1 + 4\upalpha } \right)}}. \\ \end{aligned}$$

The residual function can be obtained as:

56 $$\:\widetilde{Res}\left(x,t,\widetilde{u}\right)={\text{D}}_{\text{t}}^{{\upalpha\:}}\widetilde{u}+\left(\widetilde{u}\right){\widetilde{u}}_{x}+{\widetilde{u}}_{xxx}-{\widetilde{u}}_{xxxxx},$$

with the initial condition:

57 $$\:{\widetilde{\text{u}}}_{0}=\:\frac{105}{169}\:{\text{s}\text{e}\text{c}\text{h}}^{4}\left(\frac{\text{x}}{2\:\sqrt{13}}\right).$$

By using $\:{\widetilde{u}}_{0}$ and put $\:{c}_{0}=1,\:\widetilde{u}\:$can written as:

58 $$\begin{aligned} \widetilde{u} & = \left( {\frac{{105}}{{169}}\:sech^{4} \left( {\frac{x}{{2\:\sqrt {13} }}} \right)} \right) + c_{1} \left( {\frac{{7560\:\:sech^{4} \left( {\frac{x}{{2\sqrt {13} \:}}} \right){\text{tanh}}\left( {\frac{x}{{2\sqrt {13} \:}}} \right)}}{{28561\:\sqrt {13} }}} \right)\frac{{t^{{\alpha \:}} }}{{\Gamma \left( {1 + \upalpha \:} \right)}} \\ & \quad + \:\:c_{2} \left( {\frac{{136080\:\:sech^{6} \left( {\frac{x}{{2\sqrt {13} \:}}} \right)\left( { - 3 + 2{\text{cosh}}\left( {\frac{x}{{2\sqrt {13} \:}}} \right)} \right)}}{{62748517\:}}} \right)\frac{{t^{{2\alpha \:}} }}{{\Gamma \left( {1 + 2\upalpha \:} \right)}} \\ & \quad + \:\:\:c_{3} \left( {\frac{{4898880\:\:sech^{7} \left( {\frac{x}{{2\sqrt {13} \:}}} \right)\left( { - 13\:{\text{sinh}}\left( {\frac{x}{{2\sqrt {13} \:}}} \right) + 2{\text{sinh}}\left( {\frac{x}{{2\sqrt {13} \:}}} \right)} \right)}}{{10604499373\:\sqrt {13} \:}}} \right)\frac{{t^{{3\alpha \:}} }}{{\Gamma \left( {1 + 3\upalpha \:} \right)}} \\ & \quad + c_{4} \left( {\frac{{88179840\:\:sech^{8} \left( {\frac{x}{{2\sqrt {13} \:}}} \right)\left( {52 - 49\:{\text{cosh}}\left( {\frac{x}{{2\sqrt {13} \:}}} \right) + 4{\text{cosh}}\left( {\frac{{2x}}{{\sqrt {13} \:}}} \right)} \right)}}{{23298085122481}}} \right)\frac{{t^{{4\alpha \:}} }}{{\Gamma \left( {1 + 4\upalpha \:} \right)}}. \\ \end{aligned}$$

Hence, by substituting $\:\widetilde{u}$ into $\:\widetilde{Res}\left(x,t,\widetilde{u}\right)$, then we obtain $\:\widetilde{Res}$. Subsequently, the functional J can be expressed as:

59 $$\:{\iint\:}_{{\Omega\:}}^{.}{\widetilde{Res}\left(x,t,\widetilde{u}\right)}^{2}\:dx\:dt=\:J\left({c}_{1},{c}_{2},{c}_{3},{c}_{4}\right).$$

By evaluating the functional J, we arrive at four algebraic equations, which can be stated as follows:

60 $$\:\frac{\partial\:J}{\partial\:{c}_{1}}=\frac{\partial\:J}{\partial\:{c}_{2}}=\frac{\partial\:J}{\partial\:{c}_{3}}=\frac{\partial\:J}{\partial\:{c}_{4}}=0.$$

Subsequently, the unknown coefficients $\:\left({c}_{1},{c}_{2},{c}_{3},{c}_{4}\right)$ for the case $\:\:{\upalpha\:}=1$ take the form:

61 $$\:\begin{array}{c}{c}_{1}=1.000000000023623,{c}_{2}=0.999999986234735,\\\:{c}_{3}=1.000004426520602,{c}_{4}=0.998830217957528.\end{array}$$

The formula for absolute error is:

62 $$\:Error=\left|{\widetilde{u}}_{i}(x,t)-u(x,t)\right|.$$

## Conclusion

**Fig. 1:**
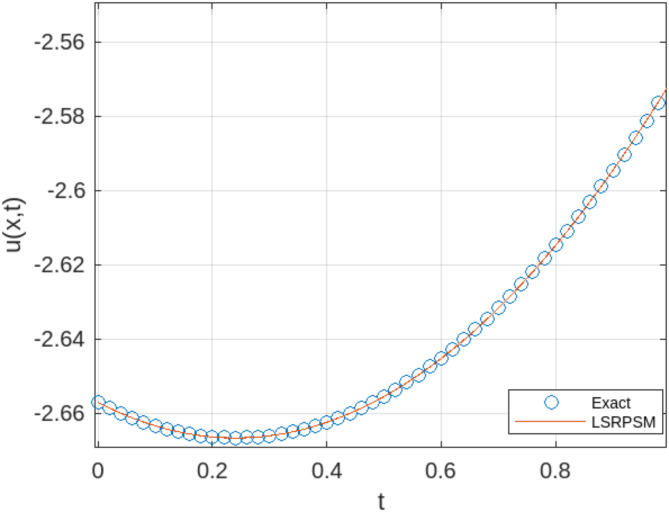
Comparison between the LSRPSM and the exact solution for the Rosenau-Hyman equation$\:\:at\:x=1$ and$\:\:\alpha\:=1$.

**Fig. 2:**
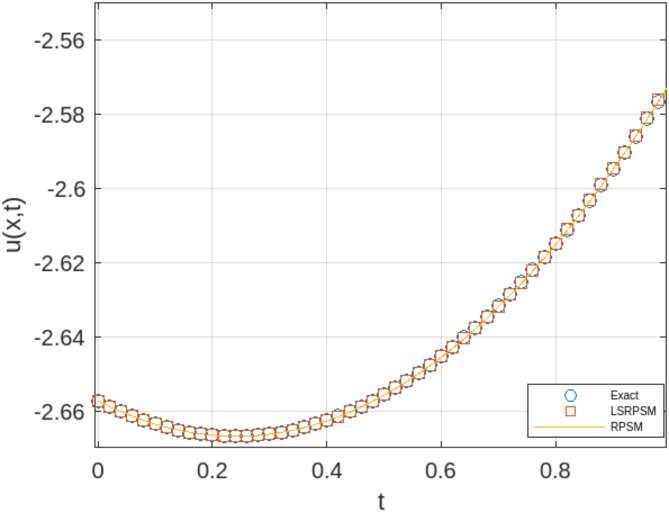
Comparison of the LSRPSM, RPSM, and exact solutions for Rosenau-Hyman equation at$\:\:x=1$.

**Fig. 3:**
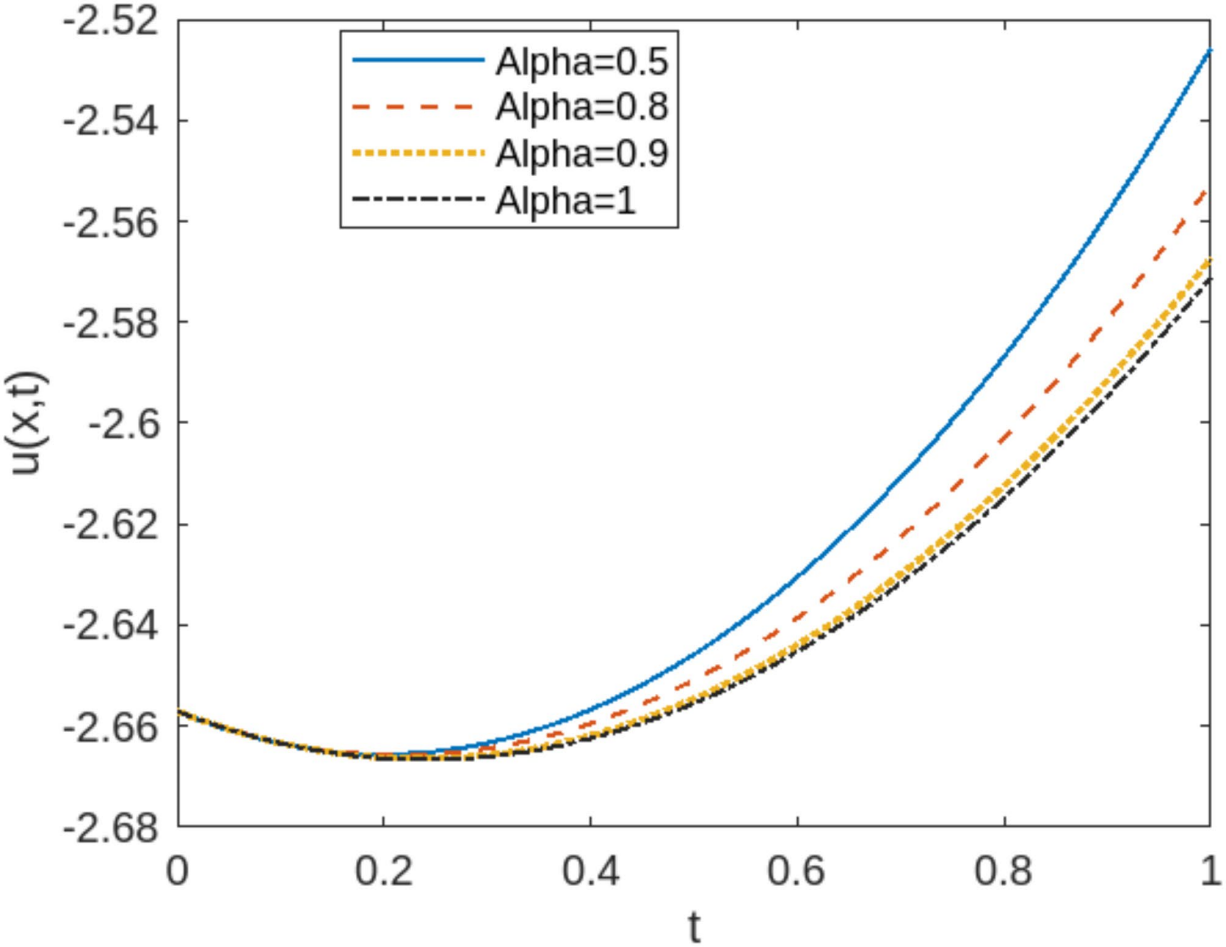
LSRPS solution for Eq. (4.17) at different values of $\:\alpha\:$.

**Fig. 4:**
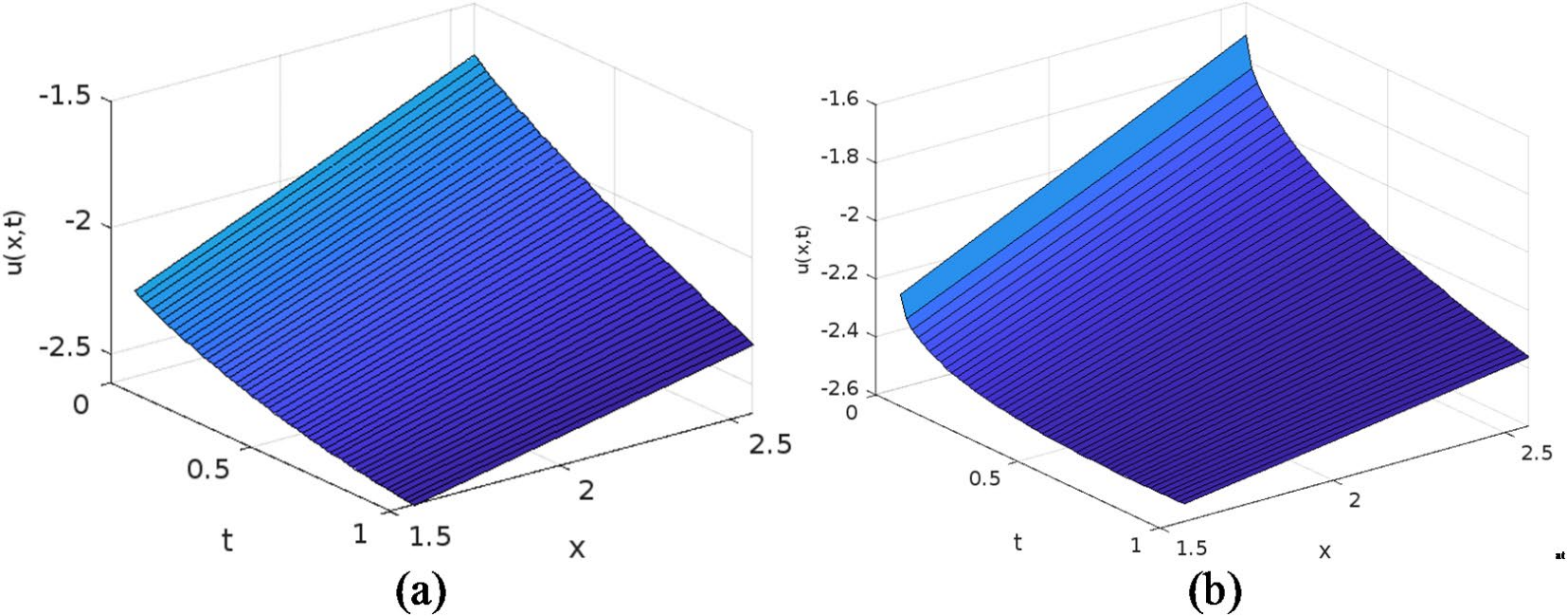
3D surface graphs of the fractional Rosenau-Hyman equation (**a**) $\:\alpha\:=0.9$ (**b**) $\:\alpha\:=0.5$.

**Fig. 5:**
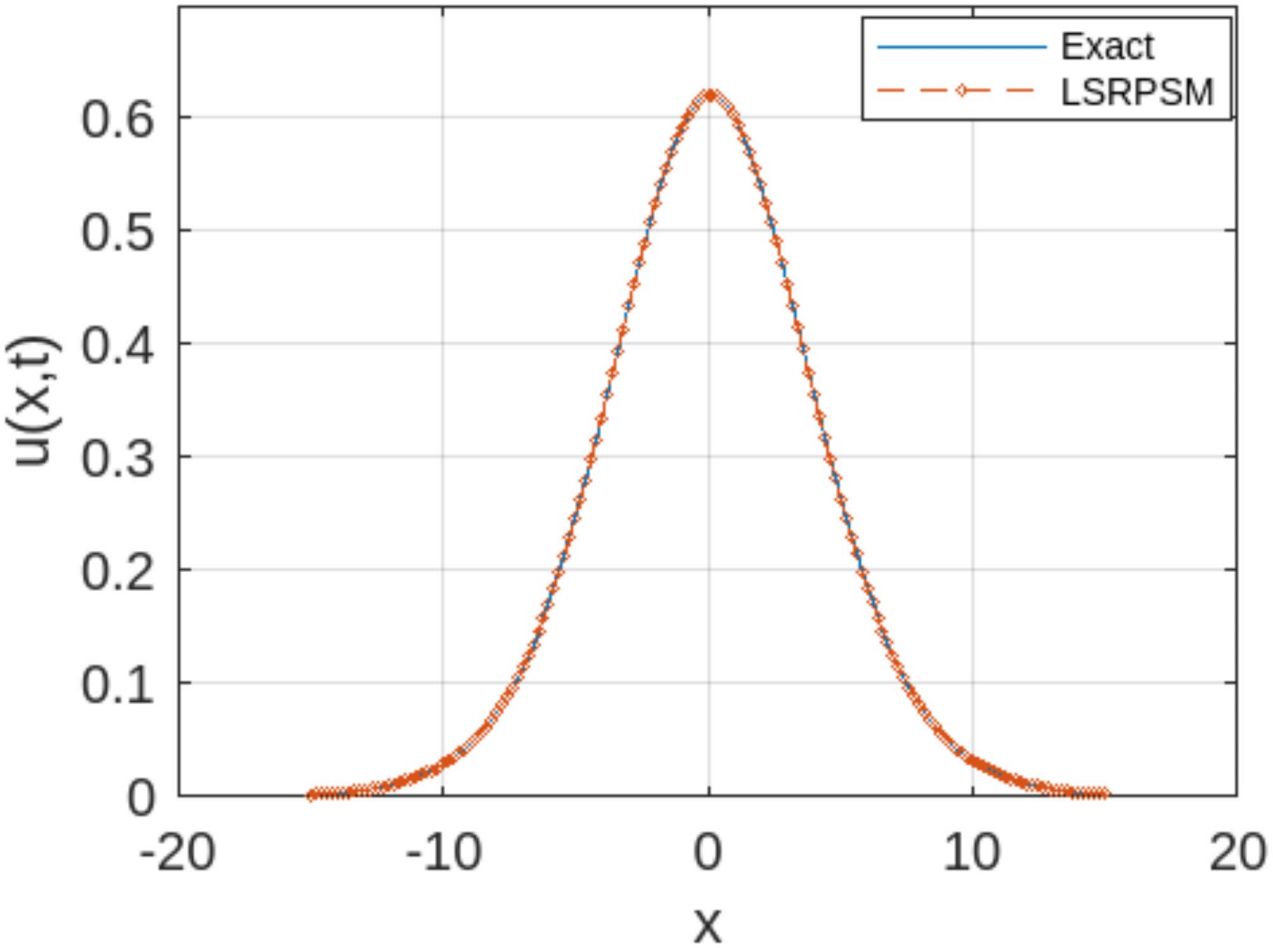
Comparison between the LSRPSM and the Exact Solutions for the Kawahara Equation at $\:x=10$.

**Fig. 6:**
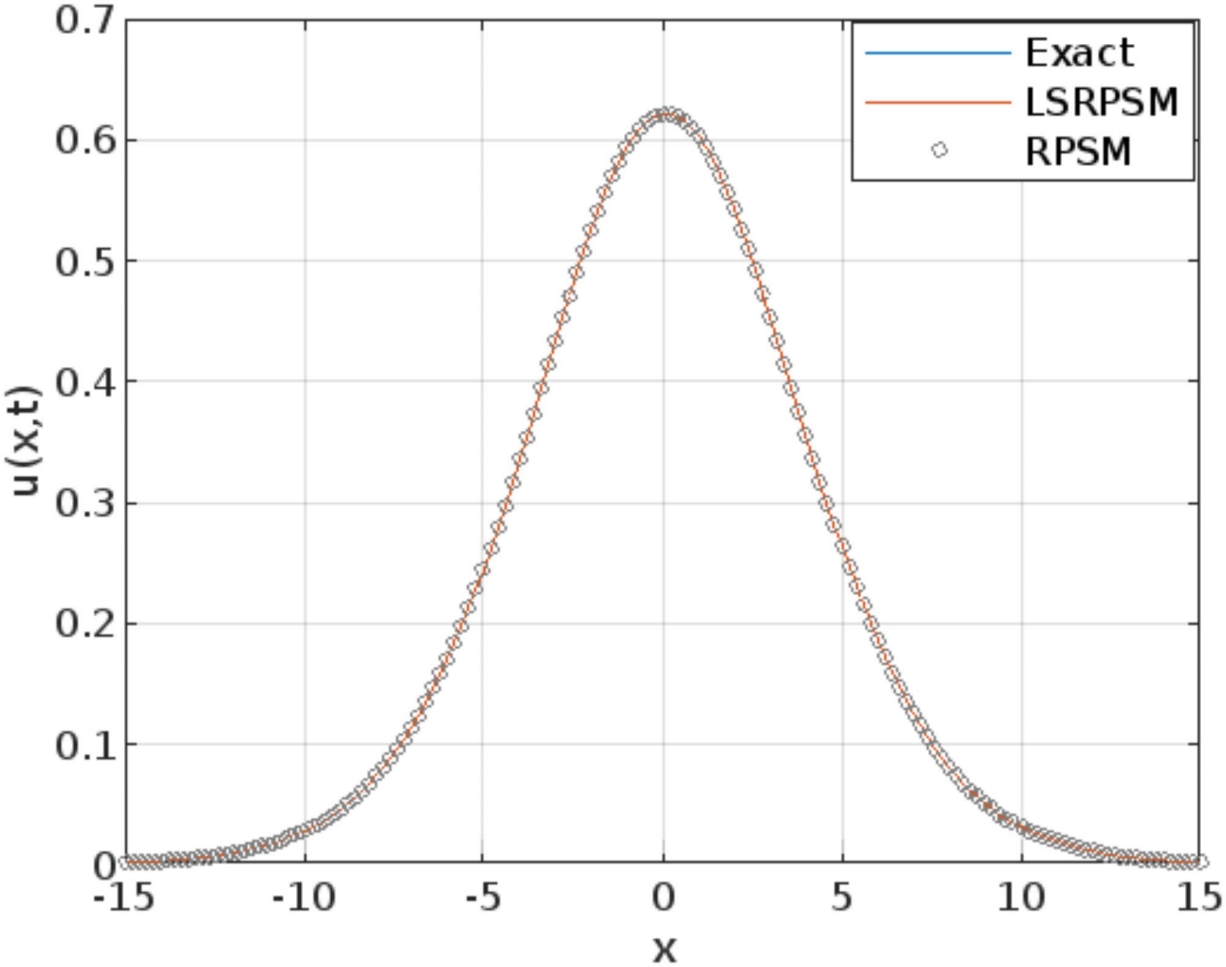
Comparison between LSRPSM, RPSM and the Exact Solutions for the Kawahara Equation at $\:x=10$.

**Fig. 7:**
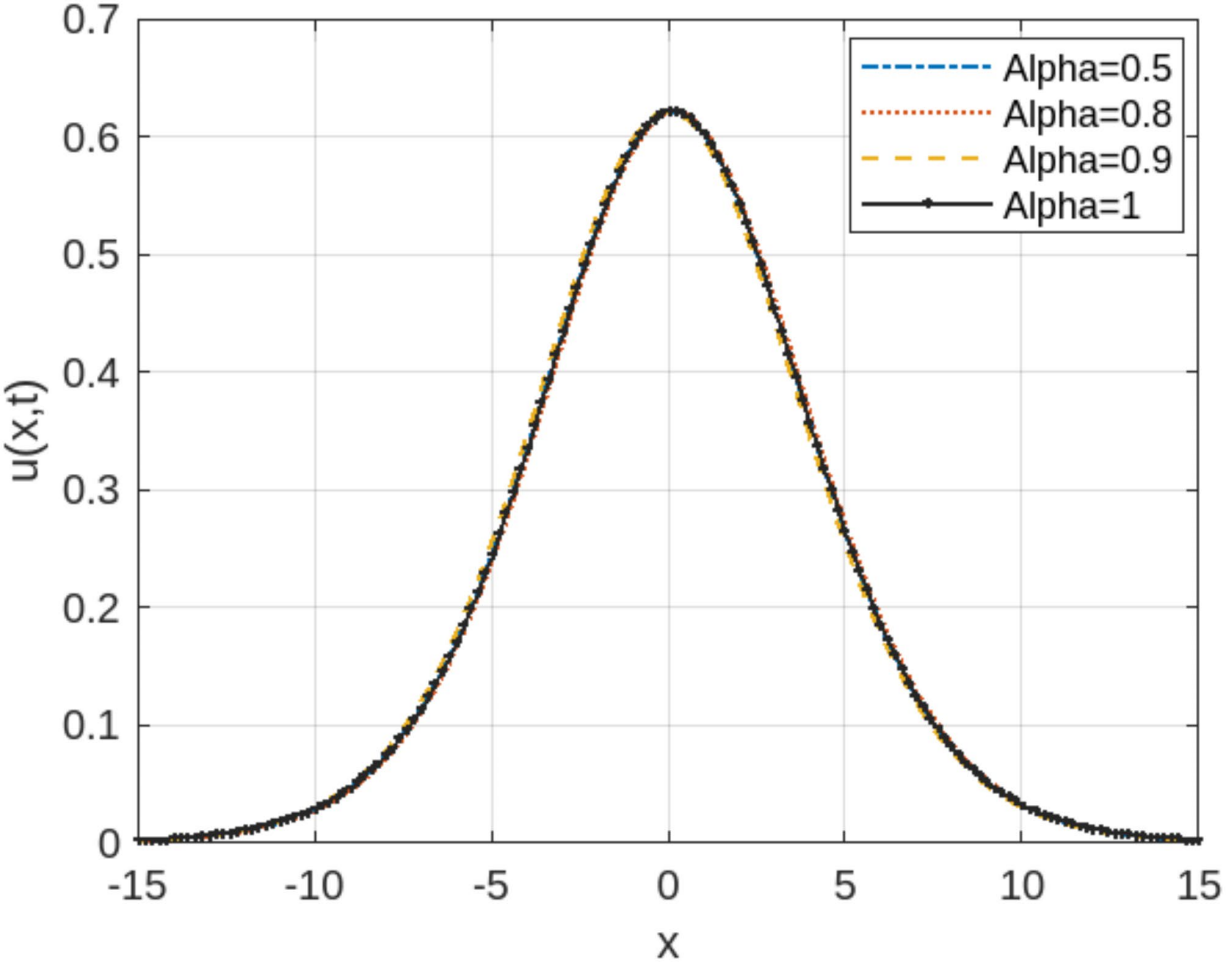
LSRPS Solution of Eq. (46) using for different values of $\:\alpha\:$.

**Fig. 8:**
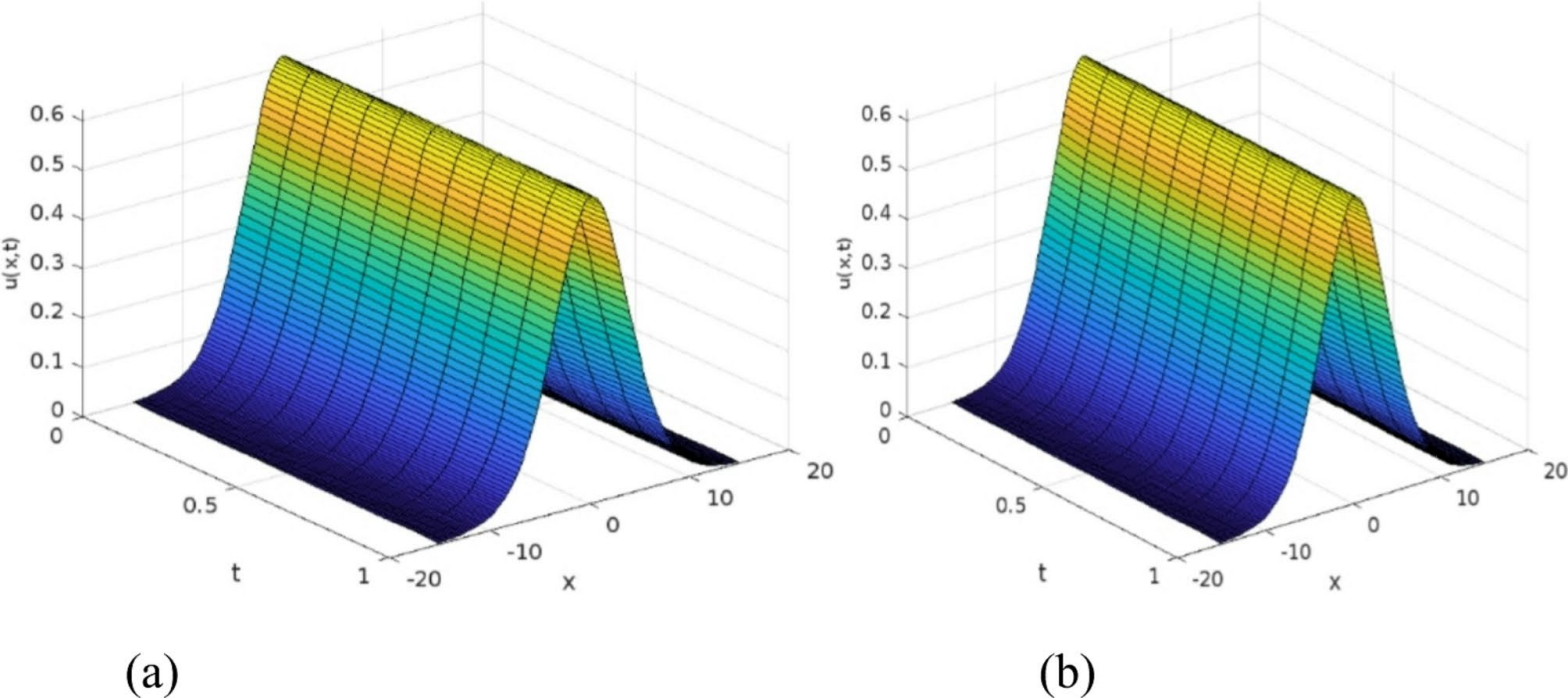
3D surface graphs of the fractional Kawahara equation (**a**) $\:\alpha\:=0.9$ (**b**) $\:\alpha\:=0.5$.

The least squares residual power series method (LSRPSM) is an improved version of the residual power series method (RPSM), incorporating the least squares technique to improve accuracy and convergence. This paper introduces a novel comparative analysis between LSRPSM and RPSM, highlighting the advantages of incorporating the least squares approach in improving accuracy and convergence speed. The correctness of these results is displayed in Tables 1 and 2 and visually in Figs. 1, 2, 3, 4, 5, 6, 7 and 8 to demonstrate the effectiveness and distinctiveness of this approach. It can be observed that the LSRPSM often converges more quickly than RPSM, especially for problems with complex boundary conditions. It is suitable for problems where standard RPSMs cannot achieve sufficient accuracy within a limited number of terms. In this regard, it is significant and a useful alternative approach for resolving fractional NPDEs. In the future, least squares technique can be combined with other analytical methods to obtain optimal results. Other fractional definitions can also be used.

**Table 1:** The absolute errors between the approximate and exact solutions obtained using the (LSRPS) approach with the (RPS) method for problem 1 at $\:\alpha\:=1$.

t	x	RPSM	LSRPSM	Exact	Absolute error LSRPSM	Absolute error RPSM^34^
0.2	$\:\frac{{\uppi\:}}{2}$	− 2.3657092	− 2.36545716	− 2.36555610	9.8936223E − 05	1.53100E − 04
0.6	$\:\frac{{\uppi\:}}{2}$	− 2.5165586	− 2.51543414	− 2.51265233	2.7818097E − 03	3.906270E − 03
1	$\:\frac{{\uppi\:}}{2}$	− 2.6296958	− 2.62720764	− 2.61273283	1.447480E − 02	1.69630E − 02
0.2	$\:{\uppi\:}$	− 1.4666666	− 1.46639701	− 1.46644455	4.7545022E − 05	2.21100E − 04
0.6	$\:{\uppi\:}$	− 1.7333333	− 1.73252436	− 1.72736027	5.164089E − 03	5.973030E − 03
1	$\:{\uppi\:}$	− 2.0000000	− 1.99865171	− 1.97256738	2.608433E − 02	2.74326E − 02

**Table 2:** Comparison between the least squares residual power series method (LSRPSM) and the residual power series method (RPSM) for problem 2 at $\:\alpha\:=1$.

t	x	LSRPS	Exact	Absolute error (LSRPSM)	Absolute error (RPSM)^18^
0	10	0.030421	0.030421	0	0
0.1		0.030739	0.030739	6.7233E − 16	1.41553E − 15
0.2		0.031061	0.031061	9.124E − 15	4.68063E − 14
0.3		0.031386	0.031386	9.9486E − 14	3.6391E − 13
0.4		0.031714	0.031714	5.4738E − 13	1.56886E − 12
0.5		0.032046	0.032046	2.0665E − 12	4.89617E − 12
0.6		0.03238	0.03238	6.0629E − 12	1.24542E − 11
0.7		0.032718	0.032718	1.4917E − 11	2.75069E − 11
0.8		0.033059	0.033059	3.2291E − 11	5.47829E − 11
0.9		0.033403	0.033403	6.3467E − 11	1.0081E − 10
1		0.033715	0.033715	1.1571E − 10	1.7428E − 10

## Data Availability

Data is provided within the manuscript or supplementary information files.
